# Unusual Product Distribution from Friedländer Reaction of Di- and Triacetylbenzenes with 3-Aminonaphthalene-2-carbaldehyde and Properties of New Benzo[*g*]quinoline-Derived Aza-aromatics

**DOI:** 10.3390/molecules190812842

**Published:** 2014-08-21

**Authors:** Moinul Karim, Yurngdong Jahng

**Affiliations:** Pharmacy, Yeungnam University, Gyeongsan 712-749, Korea

**Keywords:** Friedländer reactions, benzo[*g*]quinoline, 2-phenylbenzo[*g*]quinoline, 2-(pyrid-2-yl)benzo[*g*]quinoline, 1,3-di(benzo[*g*]quinolin-2-yl)benzene, 1,3,5-tri(benzo[*g*]quinolin-2-yl)benzene, *N*,*C*-bidentate, photoluminescence

## Abstract

The Friedländer reactions of acetylbenzenes and 2-acetylpyridine with 3-aminonaphthalene-2-carbaldehyde afforded the corresponding 2-phenylbenzo[*g*]quinoline and 2-(pyrid-2-yl)benzo[*g*]quinoline, respectively. The same reactions of 3-aminonaphthalene-2-carbaldehyde with 1,2-, 1,3-, 1,4-di- and 1,3,5-triacetylbenzenes, however, afforded a series of corresponding (benzo[*g*]quinolin-2-yl)benzenes as new *N*,*C*-bidentate and unexpected benzo[*g*]quinoline. Crystallinity, thermal properties, absorption and emission spectral properties of the products were studied.

## 1. Introduction

The 2-phenylpyridine molecule is itself a monodentate ligand, of which the initial *N*-coordinated intermediate nevertheless undergoes cyclometalation of the C-H bond at the *ortho*-position with a variety of metals, especially d^4^ and d^6^ metals, to form the common N^C-bidentate cyclometalated five-membered rings [1]. The most intriguing properties of 2-phenylpyridine, especially in the area of organic light emitting devices (OLED), result from its ability [2,3] to form iridium complexes such as [Ir(N^C)_3_] [4,5,6], [Ir(N^C)_2_L]^2+^ (L = an additional N^N- [7,8,9], N^C- [10], N^O- [11], as well as O^O-bidentates [12,13], respectively), and [Ir(N^C)(N^N^N)L]^+^ (where L is an anionic monodentate ligand) [14]. 2-Phenylquinoline [15,16] and 2-phenylbenzo[*g*]quinoline (**3aa**) [17,18] have been introduced as benzo-fused analogs of 2-phenylpyridine to improve the luminescence intensity, efficiency, and/or lifetime. However, systematic studies on the preparation of the systems with benzo[*g*]quinoline (BQ) have not been pursued as yet.

The preparation methods of **3aa** found in the literature, included a Grignard reaction of benzo[*g*]quinoline with phenylmagnesium bromide [19,20], a Pfitzinger reaction of 5,6-benzoisatin and acetone [21], a Friedländer reaction of acetophenone and 3-aminonaphthalene-2-carbaldehyde [22], and an electrophilic cyclization of 2-azido-3-(3-phenylprop-2-yn-1-yl)naphthalene [23]. The Friedländer reaction has, however, been one of the most effective methods for quinoline-based heterocycles, even though the preparation of some prerequisite *o*-aminoarenecarbaldehydes requires somewhat lengthy reaction sequence [24].

As a part of our interest in azapolyaromatics [25], we describe herein Friedländer reactions of acetylbenzene and polyacetylbenzenes with 3-aminonaphthalene-2-carbaldehyde for the synthesis of a series of benzo[*g*]quinoline-derived aza-aromatics and some properties of the resulting products.

## 2. Results and Discussion

### 2.1. Synthesis

**Scheme 1:**
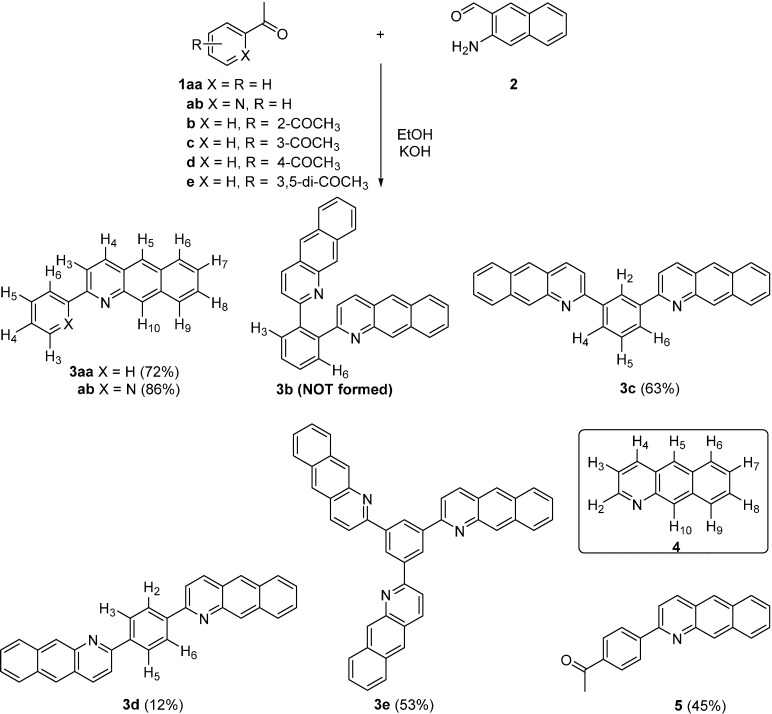
Synthesis of benzo[*g*]quinoline-derived azaaromatics.

Synthesis of the designed compounds was straightforward as shown in Scheme 1. The Friedländer reactions of a series of acetylbenzenes **1** with 3-aminonaphthalene-2-carbaldehyde (**2**) in the presence of KOH afforded the desired products **3** in 12%–72% yields, except for **1b**. Although reactions of acetylbenzene (**1aa**) and 2-acetylpyridine (**1ab**) with **2** afforded the Friedländer adducts **3aa** and **3ab** [26] in 72% and 86% yield, respectively, with a trace of as yet unidentifiable products, reactions of diacetylbenzenes **1b**, **1c** and **1d** led somewhat unexpected results. The reactions between 1,2-diacetylbenzene (**1b**) and **2** resulted in formation of benzo[*g*]quinoline (**4**) in 40% yield as a major products without any trace of the expected **3b**, while the reactions of 1,3- (**1c**) and 1,4-diacetylbenzene (**1d**) afforded the desired Friedländer products **3c** and **3d** in 63% and 12%, respectively, along with **4** in 24% and 33%, respectively. It should be noted that the reaction of **1d** with excess (2.2 equiv.) **2** afforded the monocondensed 1-(benzo[*g*]quinolin-2-yl)-4-acetylbenzene (**5**) in 45% yield, which also led to formation of **3d** and **4** in a similar ratio by subsequent Friedländer reaction with additional **2**. The structure of **4** was confirmed by physical properties and comparison to the spectral data in the literature [27]. ^1^H-NMR showed two characteristic resonances for H_2_ and H_3_ of BQ moiety as a doublet of doublets at δ 8.97 (*J*_2,3_ = 4.3, *J*_2,3_ = 1.2 Hz) for H_2_ and δ 7.35 (*J*_3,4_ = 8.5, *J*_2,3_ = 4.3 Hz) for H_3_, respectively. Although the Friedländer reaction of **1b** with 4-aminoacridine-3-carbaldehyde [28] and the reactions of triacetylmethane with *o*-aminoarenecarbaldehydes [29] resulted in similar type of unexpected products, a possible reaction mechanism for **4** remains to be clarified.

### 2.2. Spectroscopic Properties

The ligands prepared could be readily characterized by ^1^H-NMR spectral data and electrospray ionization mass spectrometry. Selected proton resonances are summarized in Table 1. Even though it was not always possible to completely resolve and assign all the proton resonances, certain features were characteristic and diagnostic enough to provide crucial clues about the structures. Typically, H_5_ and H_10_ of the benzo[*g*]quinoline (BQ) moiety and H_2_ (and/or H_6_) in the phenyl (Ph) ring of **3** are the ones to allow easy assignment by comparing their chemical shifts and splitting patterns as well as numbers of protons. In **3aa**, H_5_ and H_10_ of BQ were resonated at δ 8.65 and 8.76 as an one-proton singlet, respectively, while H_2_ and H_6_ of Ph at δ 8.36 as a two-proton doublet of doublet (*J* = 8.1, 1.2 Hz). Introducing an additional BQ moiety on benzene ring usually resulted in downfield-shift of these protons. Introduction of BQ moiety to C_3_ of the central benzene ring led to significant shift of H_2_ of Ph by 0.76 ppm resonating at δ 9.12 as a one-proton triplet (*J* = 0.8 Hz). In tri-substituted system **3e**, H_2_ of Ph was resonated at δ 9.30 as a three-proton singlet due to the two adjacent N_1_’s of BQ moiety that is comparable to those of 1,3,5-tri(azaheteroar-2-yl)benzenes [28,30].

**Table 1:** Selected proton resonances for compounds **3**, **4** and **5**.

	H_2_ of Ph	H_3_ of Ph	H_2_ of BQ	H_3_ of BQ	H_4_ of BQ	H_5_ of BQ	H_10_ of BQ
**3aa**	8.24	7.89	-	-	8.38	8.40	8.77
**3ab**	-	8.74 ^a^	-	8.57	8.46	8.44	8.79
**3c**	9.12	8.40	-	-	8.46	8.43	8.70
**3e**	9.30	8.25	-	-	8.52	8.48	8.90
**4**	-	-	8.97	7.35	8.58	8.40	8.76
**5**	8.51	8.26–8.15	-	8.26–8.15	8.689	8.69	8.81

Note: ^a^ This resonance corresponds to H_3_ of the pyridine moiety of **3ab**.

**Figure 1:**
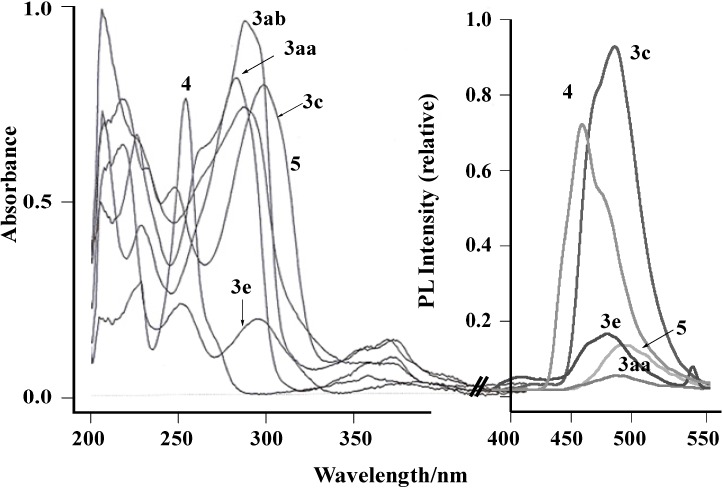
UV absorption and photoluminescence (PL) spectra of **3**, **4**, and **5** in EtOH (1 × 10^−5^ M) at 298 K. In PL spectra emission of **3ab** was overlapped with that of **5**, thus omitted for clarity.

UV absorption spectra of **3** and **5**, and the parent **4** in EtOH (1 × 10^−5^ mol/L) were investigated, and the data are given in Figure 1 and Table 2. All compounds display intense absorption bands in the ultraviolet region 205–400 nm with extinction coefficients (ε) of ~10^5^, which are assigned to spin-allowed ^1^LC transitions.

**Table 2:** UV absorption and emission spectral data of **3**, **4** and **5**.

Compound	λ_abs/nm_ (logε)	λ_excit_	λ_em_
**3aa**	205 (4.86) 228 (4.63) 257 (s, 4.60) 282 (4.91) 352 (3.81) 371 (3.96)	282	481
**3ab ^a^**	204 (4.70) 225 (4.83) 259 (s, 4.78) 287 (4.98) 351 (4.04) 370 (4.15)	225	488
**3c**	205 (5.00) 228 (s, 4.80) 265 (s, 4.76) 286 (4.86) 371 (3.88)	286	470
**3e**	209 (s, 4.78) 216 (4.81) 249 (4.36) 294 (4.28) 378 (3.43)	294	470
**4**	203 (4.30) 227 (4.46) 253 (4.88) 272 (s, 4.08) 358 (3.66)	253	435
**5**	205 (4.83) 216 (4.88) 232 (s, 4.76) 246 (4.72) 298 (4.90)	298	470

Note: ^a^ Excitation of the absorbance at 287 nm did not show any observable emission.

The photoluminescence (PL) of the compounds was studied in EtOH (1 × 10^−5^ mol/L) at room temperature and are given in Table 2. Excitation of the absorbance in the region 253–294 nm showed greenish blue light emissions in the range of 470–488 nm. The observed emission wavelength is somewhat dependent on the nature of the central benzene ring: Disubstituted ligands (**3c**, **3e**, **5**) showed emissions at 470 nm while monosubstituted ones (compounds **3aa**, **3ab**) showed them at 481 and 488 nm. The parent benzo[*g*]quinoline showed blue light emission at 435 nm. It should be noted that the emission of **3c** and **3e** were the relatively high compared to those (Figure 1).

### 2.3. Thermal and Structural Properties

**Figure 2:**
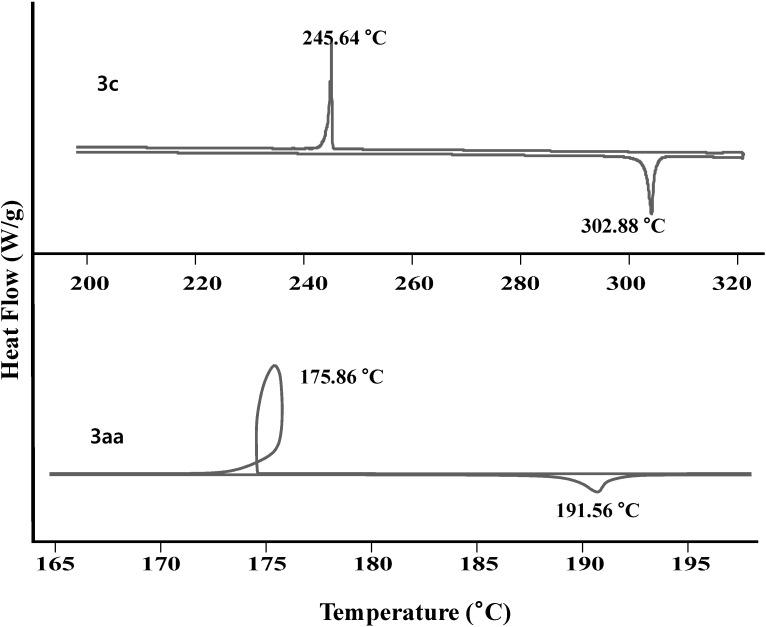
DSC of **3aa** and **3c**.

The thermal behaviors of the compounds were analyzed by differential scanning calorimetry (DSC). All the compounds showed a single sharp endothermic peak at the melting transition temperature (*T*_m_) and exothermic peaks at the crystallization temperature (*T*_c_) as shown in Figure 2. However, none of the compounds showed glass transition temperature (*T*_g_). It should be noted that compounds **3aa** and **5** showed temperature increasing during crystallization implying that super cooling may be accompanied during crystallization. As a result, all the compounds prepared have good thermal stability despite of being relatively low molecular weight organic compounds.

**Figure 3:**
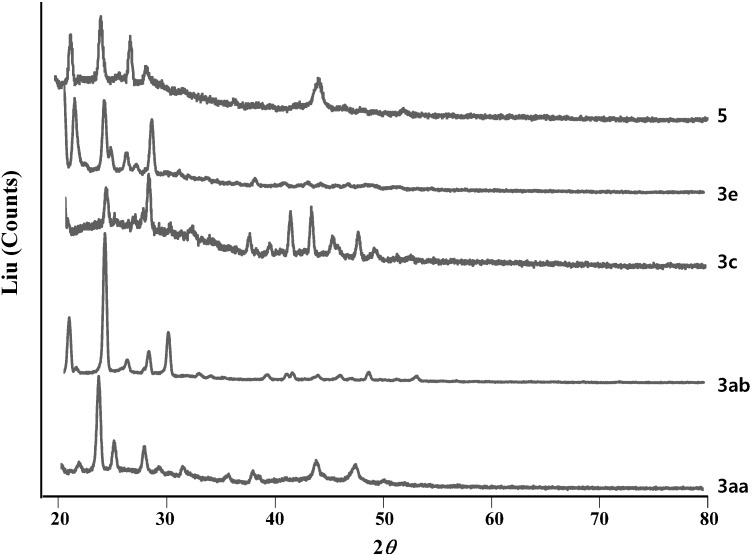
X-ray diffractograms of compounds prepared in powder state.

The crystallinity of the compounds prepared was analyzed by XRD (X-ray diffraction) and X-ray diffractograms are shown in Figure 3. All of X-ray diffractograms of the compounds showed numerous distinctive peaks indicating their crystalline nature.

## 3. Experimental Section

### General Information

Melting points were determined using a Fischer-Jones melting points apparatus and are not corrected. UV spectra were recorded on a V550 spectrophotometer (Jasco, Tokyo, Japan). IR spectra were obtained using a 1330 spectrophotometer (Perkin-Elmer, city, state abbrev if US, country). NMR spectra were obtained using a Bruker-250 spectrometer (Fällanden, Switzerland) or VNS600 FT-NMR (Varian, Australia) for ^1^H-NMR and 62.5 MHz for ^13^C-NMR and are reported as parts per million (ppm) from the internal standard TMS. Chemicals and solvents were commercial reagent grade and used without further purification. Electrospray ionization (ESI) mass spectrometry (MS) experiments were performed on a LCQ advantage-trap mass spectrometer (Thermo Finnigan, San Jose, CA, USA). Elemental analyses were taken on a Hewlett-Packard Model 185B CHN analyzer (Hewlett Packard, Littleton, MA, USA). XRD analysis was performed by X-ray Diffractometer (Model: MPD for bulk, PANalytical, Wesybrough, MA, USA) with nickel-filtered CuKα radiation (30 kV, 30 mA) at 2θ angles from 10° to 90°, a scan speed of 10°/min and a time constant of 1 s. Thermal behaviors of the compounds were analyzed using differential scanning calorimetty (DSC Q200, TA Instrument, Wilminton, NJ, USA) with 1~2 mg of sample sealed in alumina in the range of 40–385 °C increasing temperature in a rate of 10 °C/min. An empty pan was used as a reference, and the DSC baseline, temperature, and enthalpy were calibrated. Starting 3-material aminonaphthalene-2-carbaldehyde (2) was prepared employing a previously reported method [15].

*2-Phenylbenzo[g]quinoline* (**3aa**). To a solution of an equimolar of acetylbenzene (**1aa**, 70 mg, 0.58 mmol) and **2** (100 mg, 0.58 mmol) in EtOH (40 mL) was added saturated KOH in EtOH (0.5–1 mL). The resulting reaction mixture was refluxed for 15 h. Evaporation of the solvent resulted in solid material which was chromatographed on silica gel eluting with CH_2_Cl_2_. The latter fractions [*R*_f_ = 0.42, CH_2_Cl_2_:EtOAc (5:1)] afforded the desired product (106 mg, 72%): mp 193–195 °C (lit. [11a] mp 188 °C; lit. [13] mp 190–192 °C). *T*c 175.85 °C. ^1^H-NMR (600 MHz, CDCl_3_) δ 8.77 (s, 1H, H_10_ of BQ), 8.40 (s, 1H, H_5_ of BQ), 8.39 (d, 1H, *J* = 9.0 Hz, H_4_ of BQ), 8.24 (dd, 2H, *J* = 8.1, 1.2 Hz, H_2_ and H_6_ of Ph), 8.10 (dd, 1H, *J* = 7.2, 0.8 Hz, H_6_/H_9_ of BQ), 8.03 (dd, 1H, *J* = 7.2, 1.8 Hz, H_9_/H_6_ of BQ), 7.89 (d, 1H, *J* = 9.0 Hz, H_3_ of BQ), 7.57–7.46 (m, 5H, H_7_, H_8_ of BQ, H_3_, H_4_, H_5_ of Ph). ^13^C-NMR (62.5 MHz, DMSO-*d*_6_) δ 157.99, 145.25, 139.83, 138.66, 134.93, 132.48, 131.09, 130.08, 129.43, 129.33, 128.56, 127.97, 127.84, 127.65, 127.28, 126.55, 119.78.

*2-(Pyridin-2-yl)benzo[g]quinoline* (**3ab**). Pale yellow needles [86%, *R*_f_ = 0.38, CH2Cl2:EtOAc (4:1)]: mp 149–151 °C (lit.^21^ mp 145–150 °C). *T*c 113.38 °C. Unreported spectral data are as follows: ^1^H-NMR (250 MHz, CDCl_3_) δ 8.79 (s, 1H, H_10_ of BQ), 8.77 (dd, 1H, *J* = 4.8, 2.1 Hz, H_6_ of py), 8.74 (dd, 1H, *J* = 8.7, 1.2 Hz, H_3_ of py), 8.57 (d, 1H, *J* = 8.9 Hz, H_3_ of BQ), 8.46 (d, 1H, *J* = 8.7 Hz, H_4_ of BQ), 8.14–8.04 (m, 2H, H_6_ and H_9_ of BQ), 7.92 (9td, 1H, *J* = 8.0, 1.8 Hz, H_4_ of py), 7.56–7.52 (m, 2H, H_7_ and H_8_ of BQ), 7.40 (ddd, 1H, *J* = 8.0, 4.8, 1.0 Hz, H5 of py). ^1^^3^C-NMR (62.5 MHz, CDCl_3_) δ 157.05, 156.41, 149.35, 144.55, 137.25, 137.16, 134.23, 132.21, 128.72, 128.33, 127.95, 126.62, 126.58, 126.41, 126.31, 124.43, 122.21, 118.78. MS (ESI) cacld for C_18_H_13_N_2_ [M + 1] 257, found 257. Anal. calcd for C_18_H_12_N_2_C, 84.35; H, 4.72; N, 10.93. Found C, 83.69; H, 4.80; N, 11.23.

*1,2-Bis(benzo[g]quinolin-2-yl)benzene* (**3b**). The same procedure described above for **3aa** was applied to 1,2-diacetylbenzene (**1b**) to produce an as yet unidentifiable product (~30%) along with known benzo[*g*]quinoline (**4**): Pale yellow needles [40%, *R*_f_ = 0.4 (CH2Cl2:EtOAc = 3:1)]: mp 110–112 °C (lit. [17] mp 108–109 °C Spectral (^1^H- and ^13^C-NMR and IR) data were identical to those reported previously.

*1,3-Bis(benzo[g]quinolin-2-yl)benzene* (**3c**). Pale yellow needles [63%, *R*_f_ = 0.46, CH2Cl2:EtOAc (4:1)]: mp 308–311 °C. *T*c 245.64 °C. ^1^H-NMR (250 MHz, CDCl_3_) δ 9.12 (t, 1H *J* = 0.8 Hz, H2 and H5 of Ph), 8.83 (s, 2H, H_10_ of BQ), 8.46 (d, 2H, *J* = 9.0 Hz, H_4_ of BQ), 8.44 (s, 2H, H_5_ of BQ), 8.40 (dd, 2H, *J* = 7.8, 1.8 Hz, H_3_ and H_6_of Ph), 8.14–8.05 (m, 4H, H_6_ and H_9_ of BQ), 8.06 (d, 2H, *J* = 9.0 Hz, H_3_ of BQ), 7.75 (t, 1H, *J* = 7.5 Hz, H_5_ of Ph), 7.57–7.49 (m, 4H, H_7_ and H_8_ of BQ). ^13^C-NMR (62.5 MHz, CDCl_3_) δ 157.86, 145.04, 140.52, 137.43, 134.54, 132.13, 129.72, 129.13, 128.82, 128.40, 127.96, 127.28, 126.61, 126.60, 126.22, 126.00, 119.19. MS (ESI) cacld for C_32_H_21_N_2_ [M + 1] 433, found 433. Anal. calcd for C_32_H_20_N_2_C, 88.86; H, 4.66; N, 6.48. Found C, 89.09; H, 4.58; N, 6.53. Benzo[*g*]quinoline (**4**): 24%.

*1,4-Bis(benzo[g]quinolin-2-yl)benzene* (**3d**). Pale yellow needles (12%) were obtained from a reaction mixture as precipitate: mp > 300 °C. This compound is not soluble either common organic solvents or HCl and thus unable to get spectral data Anal. calcd for C_32_H_20_N_2_C, 88.86; H, 4.66; N, 6.48. Found C, 88.97; H, 4.60; N, 6.43. 1-[4-(Benzo[*g*]quinolin-2-yl)phenyl]ethan-1-one (5): Pale yellow needles [45%, *R*_f_ = 0.65 (CH2Cl2:EtOAc = 3:1)]: mp 225–227 °C. *T*c 197.73 °C. IR (KBr) υ 1678 cm^−1^. ^1^H-NMR (250 MHz, DMSO-*d*_6_) δ 8.81 (s, 1H, H_10_ of BQ), 8.69 (s, 1H, H_5_ of BQ), 8.689 (d, 1H, *J* = 9.0 Hz, H_4_ of BQ), 8.51 (d, 2H, *J* = 8.8 Hz, H_2_ and H_6_ of Ph), 8.26–8.15 (m, 5H, H_3_ and H_5_ of Ph, H_3_, H_6_, and H_9_ of BQ), 7.62–7.59 (m, 2H, H_7_ and H_8_ of BQ), 2.67 (s, 3H). MS (ESI) cacld for C_21_H_15_NO [M + 1] 298, found 298. Anal. calcd for C_21_H_15_NOC, 84.82; H, 5.08; N, 4.71. Found C, 84.59; H, 5.14; N, 4.78. Benzo[*g*]quinoline (**4**): 33%.

*1,3,5-Tris(benzo[g]quinolin-2-yl)benzene* (**3e**). Pale yellow needles [53%, *R*_f_ = 0.4, CH2Cl2: EtOAc (3:1)]: mp 352–354 °C. *T*c 284.23 °C. ^1^H-NMR (250 MHz, CDCl_3_) δ 9.31 (s, 3H, H_2_, H_4_, and H_6_ of Ph), 8.90 (s, 3H, H_10_ of BQ), 8.52 (d, 3H, *J* = 8.7 Hz, H_4_ of BQ), 8.47 (s, 3H, H_5_ of BQ), 8.25 (d, 3H, *J* = 8.8 Hz, H_3_ of BQ), 8.15–8.06 (m, 6H, H_6_ and H_9_ of BQ), 7.56–7.53 (m, 6H, H_7_ and H_8_ of BQ). MS (ESI) cacld for C_45_H_28_N_3_ [M + 1] 610, found 610. Anal. calcd for C_45_H_27_N_3_C, 88.64; H, 4.46; N, 6.89. Found C, 88.89; H, 4.38; N, 6.79. Benzo[*g*]quinoline (**4**): 28%.

## 4. Conclusions

In conclusion, (benzo[*g*]quinolin-2-yl)benzene, 2-(benzo[*g*]quinolin-2-yl)pyridine, 1,3-di- and 1,3,5-tri(benzo[*g*]quinolin-2-yl)benzenes were prepared by Friedländer reactions of 3-aminonaphthalene-2-carbaldehyde with the corresponding acetylbenzenes and 2-acetylpyridine. All compounds display three intense absorption bands in the ultraviolet region (205–400 nm) with extinction coefficients (ε) of ~10^5^. Excitation of the absorbance in the region 253–294 nm showed greenish blue light emissions in the range of 470–488 nm. All the compounds showed crystalline nature and good thermal stabilities. Studies on the formation of Ir complexes and their properties are in progress and will be reported in the near future.

## Supplementary Materials

Supplementary materials (^1^H- and ^13^C-NMR spectra of the selected compounds) can be accessed at: http://www.mdpi.com/1420-3049/19/8/12842/s1.

## Supplementary Files

Supplementary File 1

## References

[1] Evans J.C.W., Allen C.F.H. (1938). 2-Phenylpyridine. Org. Syn..

[2] Taiju T., Wei H. (2014). Recent advances in multicolor emission and color tuning of heteroleptic iridium complexes. Isr. J. Chem..

[3] Zhou G., Wong W.-Y., Yang X. (2011). New design tactics in OLEDs using functionalized 2-phenylpyridine-type cyclometallates of iridium(III) and platinum(II). Chem. Asian J..

[4] Tamayo A.B., Alleyne B.D., Djurovich P.I., Lamansky S., Tsyba I., Ho N.N., Bau R., Thompson M.E. (2003). Synthesis and characterization of facial and meridional Tris-cyclometalated iridium(III) complexes. J. Am. Chem. Soc..

[5] Tsuboyama A., Iwawaki H., Furugori M., Mukaide T., Kamatani J., Igawa S., Moriyama T., Miura S., Takiguchi T., Okada S. (2003). Homoletptic cyclometalated iridium complexes with highly efficient red phosphorescence and application to organic light-emitting diode. J. Am. Chem. Soc..

[6] Baranoff E., Yum J.-H., Graetzel M., Nazeeruddin M.K. (2009). Cyclometallated iridium complexes for conversion of light into electricity and electricity to light. J. Organomet. Chem..

[7] Lowry M.S., Bernhard S. (2006). Synthetically tailored excited states: Phosphorescent, cyclometalated iridium(III) complexes and their applications. Chem. Eur. J..

[8] Yang C.-H., Cheng Y.-M., Chi Y., Hsu C.-J., Fang F.-J., Wong K.-T., Chou P.-T., Chang C.-H., Tsai M.-H., Wu C.-C. (2007). Blue-emitting heteroleptic iridium(III) complexes suitable for high-efficiency phosphorescent OLEDs. Angew. Chem. Int. Ed..

[9] Ulbricht C., Beyer B., Friebe C., Winter A., Schubert U.S. (2009). Recent development in the application of phosphorescent iridium(III) complex systems. Adv. Mater..

[10] Igarashi T., Kimura K., Nii K. (2001). Light-Emitting Material Comprising Orthometalated Iridium Complex, Light-Emitting Device, High Efficiency Red Light-Emitting Device, and Novel Iridium Complex. U.S. Patent Application.

[11] You Y., Huh J.O., Kim K.S., Lee S.W., Kim D., Park S.Y. (2008). Comment on “aggregation-induced phosphorescent emission (AIPE) of iridium(III) complexes”: Origin of the enhanced phosphorescence. Chem. Commun..

[12] Adachi C., Baldo M.A., Thompson M.E., Forrest S.R. (2001). Nearly 100% internal phosphorescence efficiency in an organic light-emitting device. J. Appl. Phys..

[13] Wu H., Yang T., Zhao Q., Zhou J., Li C., Li F. (2011). A cyclometalated iridium(III) complex with enhanced phosphorescence emission in the solid state (EPESS): Synthesis, characterization and its application in bioimaging. J. Chem. Soc. Dalton Trans..

[14] Chirdon D.N., Transue W.J., Kagalwala H.N., Kaur A., Maurer A.B., Pintauer T., Bernhard S. (2014). [Ir(N^N^N)(C^N)L]^+^: A new family of luminophores combining tunability and enhanced photostability. Inorg. Chem..

[15] Lamansky S., Djurovich P., Murphy D., Abdel-Razzaq F., Kwong R., Tsyba I., Bortz M., Mui B., Bau R., Thompson M.E. (2001). Synthesis and characterization of phosphorescent cyclometalated iridium complexes. Inorg. Chem..

[16] Shin I.-S., Kim J.I., Kwon T.-H., Hong J.-I., Lee J.-K., Kim A. (2007). Efficient electrogenerated chemiluminescence from bis-cyclometalated iridium(III) complexes with substituted 2-phenylquinoline ligands. J. Phys. Chem..

[17] Qiao J., Duan L., Tang L., He L., Wang L., Qiu Y. (2009). High-efficiey orange to near-infrared emissions from bis-cyclometalated iridium complexes with phenyl-benzoquinoline isomers as ligands. J. Mater. Chem..

[18] Zhang G., Zhang H., Gao Y., Tao R., Xin L., Yi J., Li F., Liu W., Qiao J. (2014). Near-infrared-emitting iridium(III) complexes as phosphorescent dyes for living cell imaging. Organometallics.

[19] Etienne A. (1944). Action of phenylmagnesium bromide on 1-azaanthracene. Comptes Rend..

[20] Bergstrom F.W., McCallister S.H. (1930). The preparation of 2-alkyl and 2-aryl pyridines and quinolines by the Grignard reaction. J. Am. Chem. Soc..

[21] Staehelin A. (1951). Synthesis of α-azaanthracenes starting from linear benzisatin. Comptes Rend..

[22] Yao M., Inoue H., Yoshioka N. (2005). Novel aromatic *N*-oxyl radical based on the benzo[*g*]quinoline skeleton: Synthesis and intermolecular ferromagnetic interaction. Chem. Phys. Lett..

[23] Huo Z., Gridnev I.D., Yamamoto Y. (2010). A method for the synthesis of substituted quinolones via electrophilic cyclization of 1-azido-2-(2-propynyl)benzenes. J. Org. Chem..

[24] Taffarel E., Chirayil S., Thummel R.P. (1994). Synthesis and properties of ligands based on benzo[*g*]quinoline. J. Org. Chem..

[25] Liang J., Cha H., Jahng Y. (2013). Synthesis and properties of annulated 2-(azaar-2-yl)-and 2,2'-di(azar-2-yl)-9,9'-spirobifluorens. Molecules.

[26] Haginiwa J., Higuchi Y., Ikeda S. (1979). Reactions concerned in tertiary amine *N*-oxides XIII. reactions of benzo[*f*, *h* and *g*]quinoline and acridine with aromatic amine *N*-oxides. Yakugaku Zasshi.

[27] Krapcho A.P., Gilmor T.P. (1999). General preparative route to benzo[*g*]quinoline (1-azaanthracenes). J. Heterocycl. Chem..

[28] Rahman A.F.M.M., Jahng Y. (2007). Synthesis and properties of benzo[*b*]-1,10-phenanthrolines and their ruthenium(II) complexes. Heteroat. Chem..

[29] Rahman A.F.M.M., Kwon Y., Jahng Y. (2005). Friedländer reactions of triacetylmethane: Unusual distribution of products. Heterocycles.

[30] Huu-Hoi N.P., Perin F., Jacquignon P. (1965). Nitrogen heterocyclic analogs of polyaryls. J. Heterocycl. Chem.

