# Supplementary material for: Unusual Product Distribution from Friedländer Reaction of Di- and Triacetylbenzenes with 3-Aminonaphthalene-2-carbaldehyde and Properties of New Benzo[g]quinoline-Derived Aza-aromatics
Source: Molecules. 2014 Aug 21;19(8):12842–51. doi: 10.3390/molecules190812842 (PMC6271429; doi:10.3390/molecules190812842)

Supplementary Materials

Figure S1. ^{1}H-NMR of **3ab**.

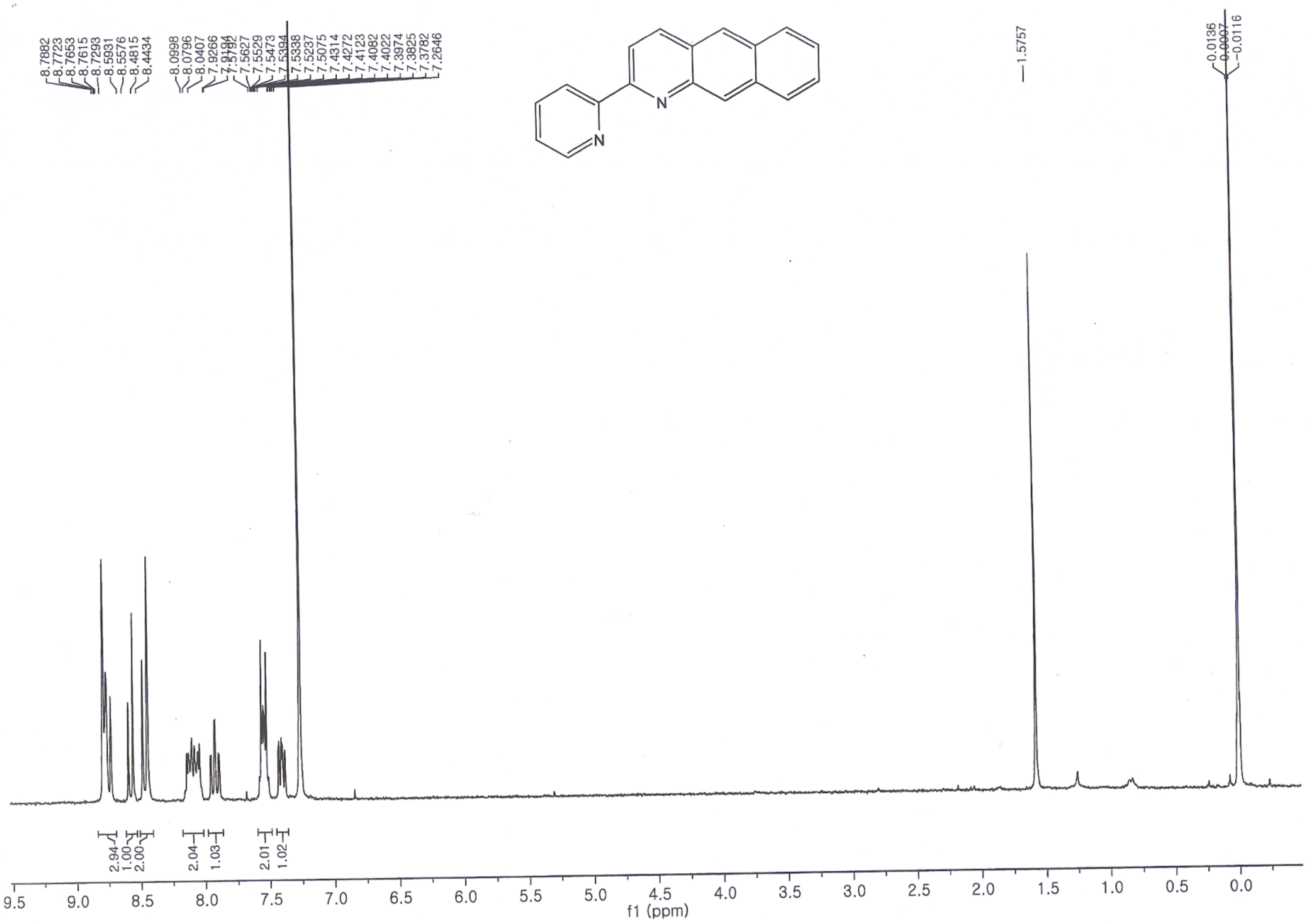

Figure S2. ^{13}C-NMR of **3ab**.

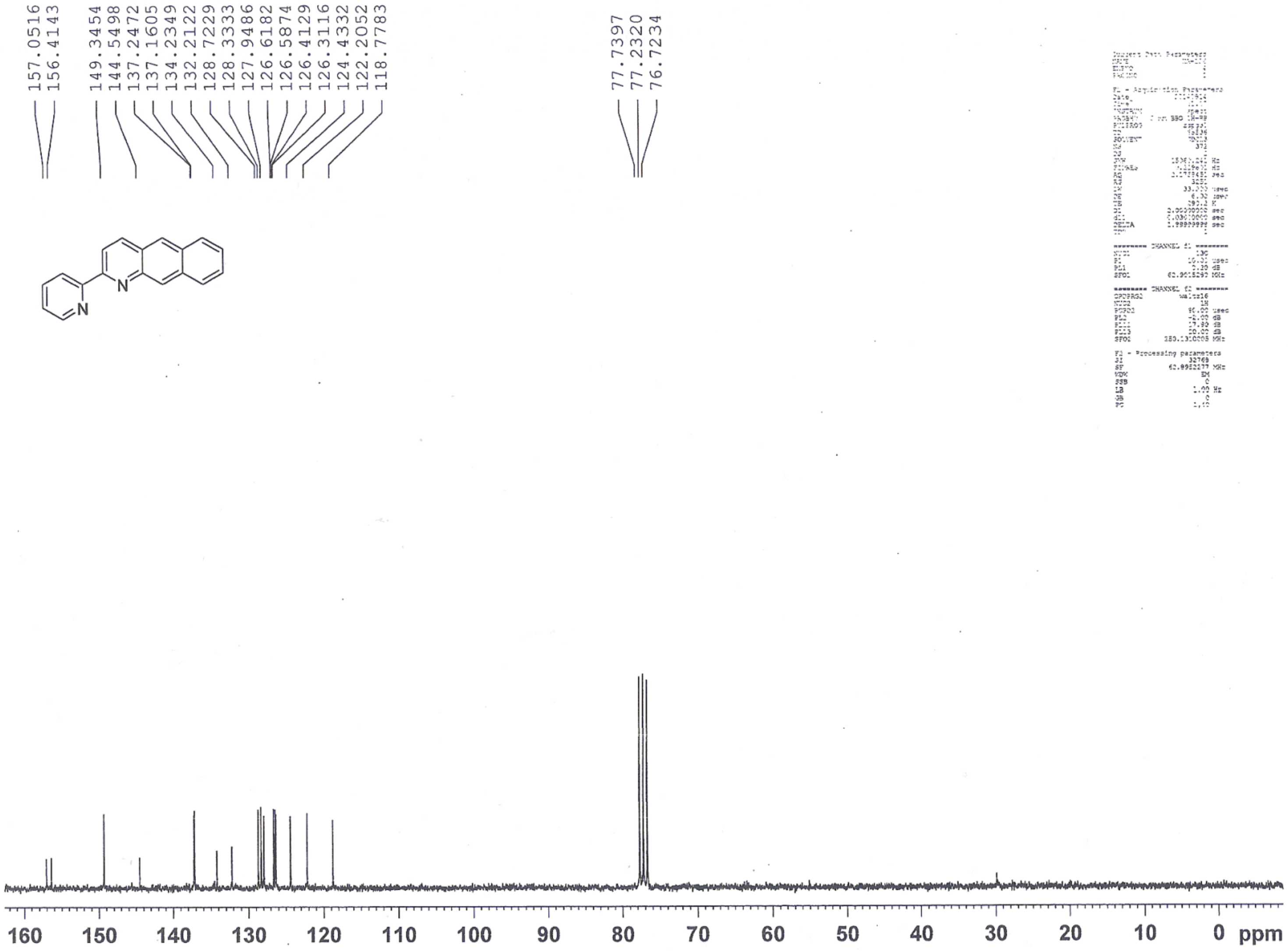

Figure S3. ^{1}H-NMR of **3c**.

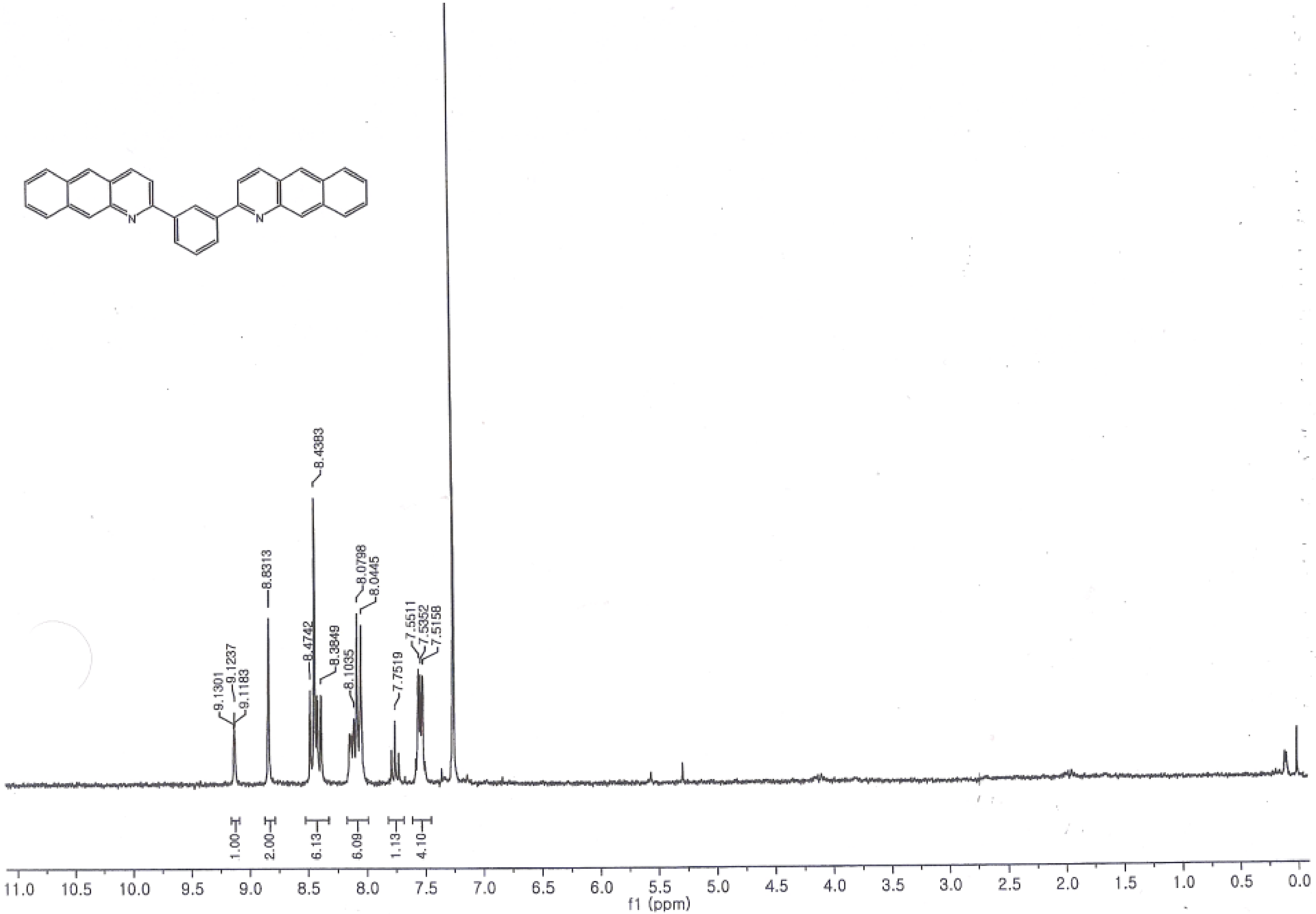

Figure S4. ^{13}C-NMR of **3c**.

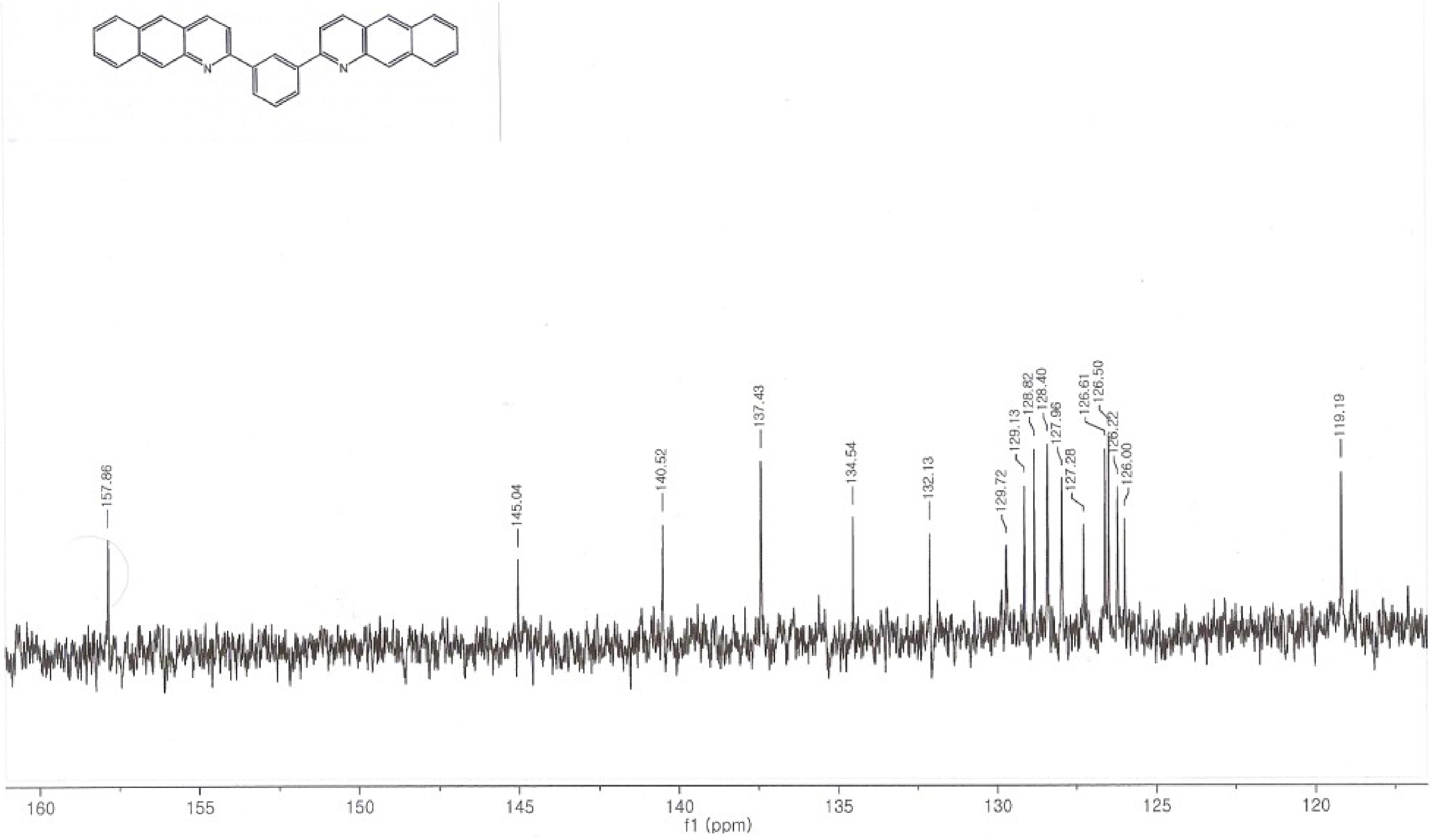

Figure S5. ^{1}H-NMR of **3e**.

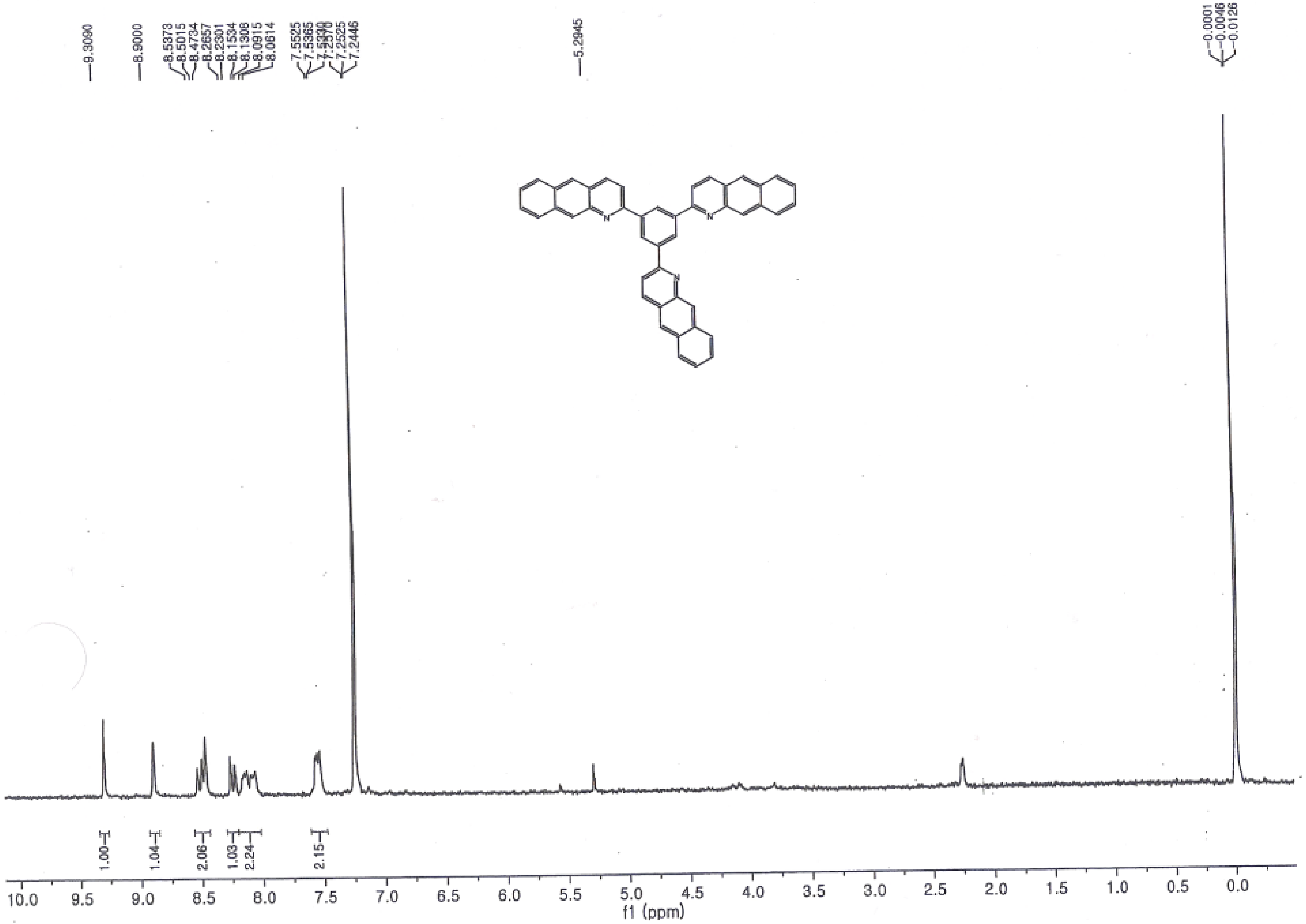

Figure S6. ^{1}H-NM0R of **4**.

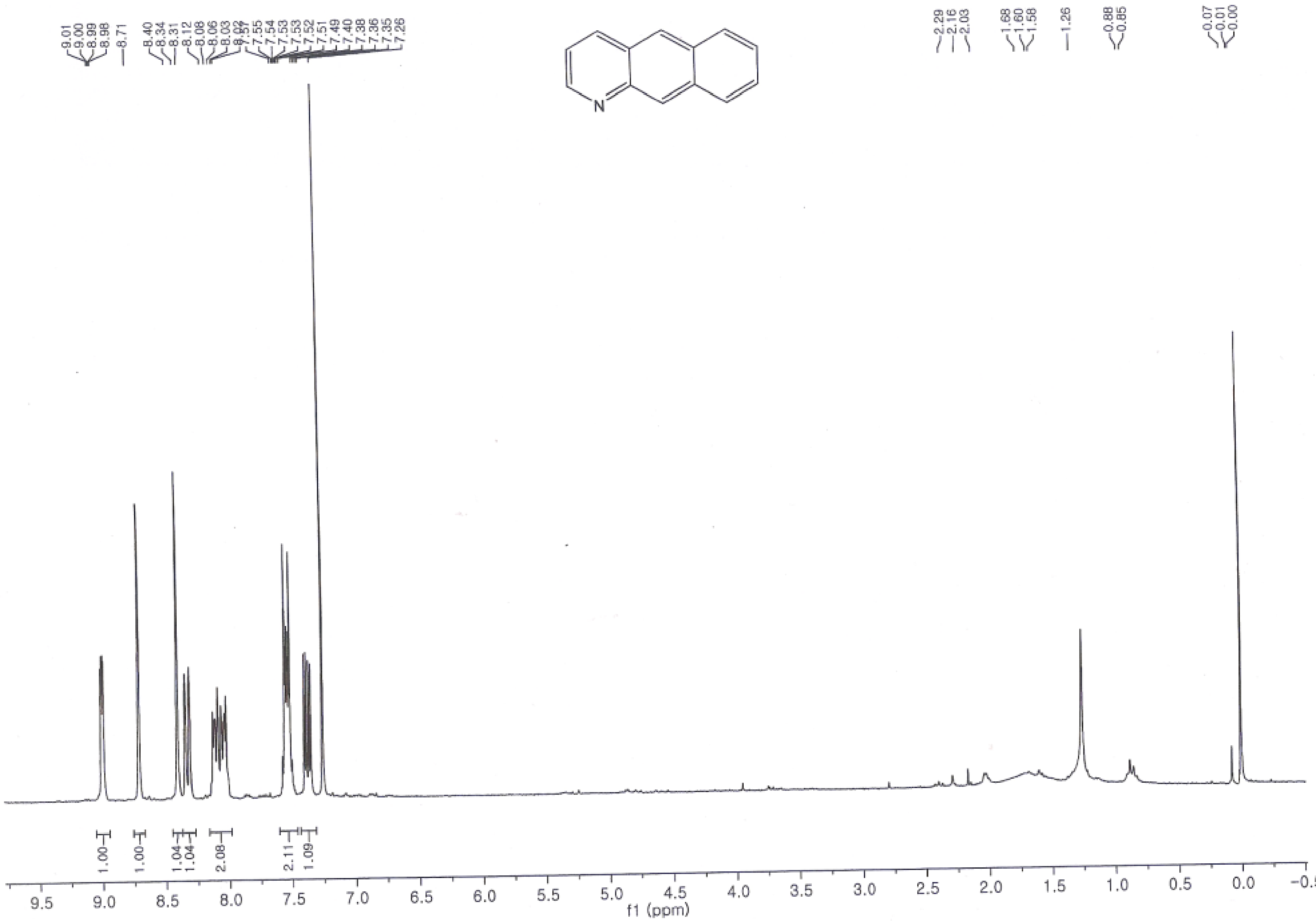

Supplement: Supplementary File 1 [file molecules-19-12842-s001.pdf]
